# Case report: Wernicke–Korsakoff syndrome after bariatric surgery

**DOI:** 10.3389/fnut.2023.1321275

**Published:** 2024-01-05

**Authors:** Margarida Alves Bento, João Barriga Vieira, Maria Leonor Silva, José Camolas

**Affiliations:** ^1^Egas Moniz School of Health and Science, Almada, Portugal; ^2^Serviço de Endocrinologia, Hospital Santa Maria, Lisbon, Portugal; ^3^Egas Moniz Center for Interdisciplinary Research, Egas Moniz School of Health and Science, Almada, Portugal; ^4^GENA (Applied Nutrition Research Group), Egas Moniz School of Health and Science, Almada, Portugal; ^5^Laboratório de Nutrição, Faculdade de Medicina, Universidade de Lisboa, Lisbon, Portugal; ^6^EnviHeB Lab, Instituto de Saúde Ambiental, Faculdade de Medicina, Universidade de Lisboa, Lisbon, Portugal

**Keywords:** Wernicke–Korsakoff syndrome, bariatric surgery, thiamine deficiency, nutritional status, sleeve gastrectomy, obesity

## Abstract

Vitamin and mineral deficiencies are prevalent nutritional disorders following bariatric surgery. Although they are more prevalent after malabsorptive procedures such as bypass, they also occur in restrictive procedures such as gastric sleeve. The mechanisms that lead to the occurrence of these deficits are related to the presence of poor nutritional intake or poor adherence to multivitamins and multimineral supplementation. Wernicke–Korsakoff syndrome (WKS) is an acute neurological disorder resulting from thiamine deficiency. This syndrome is composed of two distinct phases: first, Wernicke Encephalopathy (WE), the acute phase of this syndrome, which is characterized by a triad of mental confusion, ocular signs, and ataxia, followed by the chronic phase of WKS, called Korsakoff’s syndrome (KS), which is known for the presence of anterograde amnesia and confabulation. We aimed to report a case of a patient with WKS after bariatric surgery. The patient’s retrospective chart review was performed in order to retrieve the relevant clinical data. The patient was a 24-year-old female student with a BMI of 48 kg/m^2^ who underwent sleeve gastrectomy surgery for morbid obesity. Over the following 2 months, recovery from surgery was complicated by non-specific symptoms such as nausea, recurrent vomiting, and a significant reduction in food intake, which led the patient to visit the emergency department six times with hospitalization on the last occasion for a definitive diagnosis. During the 15 days of hospitalization, the patient developed ocular diplopia, nystagmus, complaints of rotatory vertigo, and gait abnormalities. A magnetic resonance imaging of the head was performed but revealed no significant changes. After a formal neurological assessment, treatment with parenteral thiamine (100 mg, three times a day) was started without prior dosing. The observed clinical improvement confirmed the diagnosis of WKS. Bariatric surgery may contribute to thiamine deficiency and, consequently, to WKS. Education about the adverse consequences of malnourishment is mandatory before and after the surgery. Investigation of nutritional deficiencies both pre- and post-operatively is crucial in order to prevent complications such as WKS.

## 1 Introduction

Obesity is a complex, multifactorial, adiposity-based, chronic disease defined by excessive adiposity and determined by altered and pathological mechanical forces (fat mass disease), as well as deranged endocrine and immune responses (sick fat disease) (1). This situation has been associated with an increased risk of many non-communicable diseases (1, 2). According to the World Health Organization (WHO), overweight affects approximately 60% of adults and approximately one in three children in the WHO European Region. Morbid obesity influences the population’s morbidity, mortality, and quality of life (3).

Bariatric surgery (BS) has been recommended as an effective treatment for severe obesity (4) and has increased in recent years. Surgical treatment is indicated in patients with a body mass index (BMI) higher than 40 kg/m^2^ or higher than 35 kg/m^2^ and associated comorbidities (5). In addition to weight reduction, BS has shown several benefits in obesity-related conditions, such as the management of type 2 diabetes, control of hypertension, cardiovascular benefits, reduction of obstructive sleep apnoea, reduction of disability, and reduction of health care costs (6). Among weight loss surgeries, sleeve gastrectomy (SG) is common in morbid obesity. During this surgery, a portion of the stomach is removed, resulting in restricted food consumption and reduced ghrelin secretion, promoting weight loss (7). BS has been shown to develop some complications due to the surgical procedure, such as bleeding, infection, and nutritional deficiency (3).

Wernicke–Korsakoff syndrome (WKS) is an acute neurological disorder resulting from thiamine deficiency and a possible adverse complication of BS (4). This syndrome is composed of two distinct phases: first, Wernicke Encephalopathy (WE) an acute phase characterized by a clinical triad of mental confusion, ophthalmoplegia, and ataxia (8). The full triad has been reported in less than 30% of cases, and its absence does not exclude the possibility of WE (9). The acute phase is followed by the chronic phase of WKS, known as Korsakoff’s syndrome (KS) and characterized by the presence of anterograde amnesia and confabulation (8). KS may affect up to 85% of WE patients. At least 60% of patients who survive WE also have residual physical disabilities such as nystagmus and ataxia (10).

BS presents a risk factor for WKS due to the prevalence of vitamin and mineral deficiencies following surgery. The mechanisms leading to these deficits are prevalent among patients undergoing obesity surgery and are related to the presence of poor nutritional intake and/or poor adherence to multivitamin and multimineral supplementation (4). WKS associated with BS is considered rare and can be missed. If thiamine is administered parenterally early in the course of the disease, the patient’s symptoms improve; however, if therapy is delayed, thiamine deficiency can be fatal (11). We report a case study of WKS caused by BS.

## 2 Case description

We report a case study of a 24-year-old female student affected by morbid obesity, with the following anthropometric parameters: weight 128 kg, height 163 cm, and BMI 48 kg/m^2^. Her medical history included hypothyroidism and depressive disorders. During the 2 years prior to the surgery, the patient was followed up in psychology, endocrinology, and nutrition consultations and recorded an increase in body weight of 16 kg. The patient reported no alcohol or drug use and underwent bariatric surgery.

After surgery, no early complications were observed, and the patient was discharged after 72 h with a prescription for a liquid hypo-energetic diet, which was not well tolerated with associated vomiting. The patient also showed poor adherence to multivitamin and multimineral supplementation. Over the following 2 months, the patient lost 17 kg of weight, which resulted in a BMI reduction to 42 kg/m^2^. The patient presented with generalized weakness and progressive non-specific symptoms such as nausea, recurrent vomiting, and a significant decrease in food intake. After admission to the emergency department, the patient was hospitalized for potassium replacement, but there was no improvement in diet tolerance. On another admission to the emergency department, the patient was readmitted for the exclusion of complications from the surgery.

On readmission, the neurological signs presented consisted of ocular diplopia, predominated horizontal nystagmus, complaints of rotatory vertigo, gait abnormalities, delirium with impaired orientation, and short-term recall with confabulation. On examination, her vital signs were stable, with a heart rate of 75 beats/min and blood pressure of 117/68 mm Hg. The temperature was 37.1°C. Laboratory tests revealed a glycaemia of 112 mg/dL, hypokalaemia (3.3 mmol/L), which could explain some muscle weakness, low serum osmolarity (270 mOsm/kg), and low urea (13 mg/dL), probably due to her low-protein diet. Alanine aminotransferase (ALT) was 170 U/L and aspartate aminotransferase (AST) was 61 U/L. C-reactive protein was 5.98 mg/dL, indicating some degree of infection.

The initial diagnosis of peripheral vertigo was clarified with an MRI scan of the head, which revealed no significant changes. After a formal neurological assessment, the diagnostic hypothesis of WKS was considered. This hypothesis was in line with Caine’s operational criteria for diagnosing WE, which require two of the following four signs: (a) dietary deficiencies, (b) oculomotor abnormalities, (c) cerebellar dysfunction, and (d) either an altered mental state or mild memory impairment (12). Treatment with parenteral thiamine was started without prior dosing. On the 10th day of hospitalization, the patient received intravenous thiamine (100 mg, three times a day). Three days after starting thiamine, the clinical improvement observed confirmed the diagnosis of WKS.

**Figure 1:**
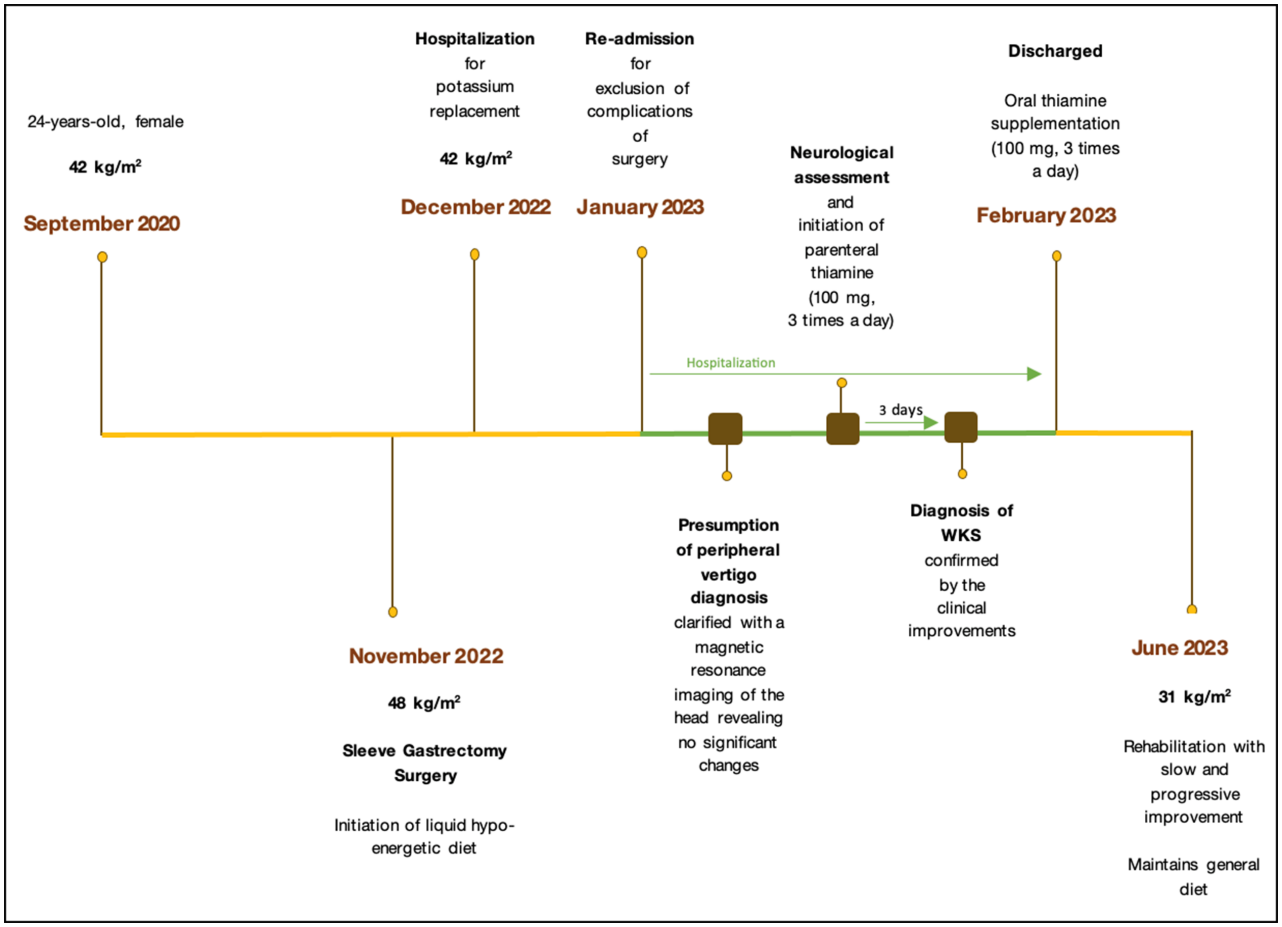
Timeline of clinical findings and nutritional intervention.

Fifteen days after hospitalization, the patient was discharged, showing signs of progressive recovery, and was instructed to continue oral thiamine supplementation (100 mg, three times a day). Over the following weeks, the patient underwent rehabilitation with slow and progressive improvement. Seven months after surgery, the patient weighs 72 kg (BMI 31 kg/m^2^) and maintains a stabilization diet without difficulty. The dose of thiamine supplementation was reduced from three times a day to one time a day due to the return of serum thiamine levels to normal values. Despite her recovery, the patient continues to have some physical limitations, such as an abnormal gait, aided by crutches, and mental confusion. The timeline of the case report is shown in Figure 1.

## 3 Discussion

Gastric resection for BS has been associated with macro- and micronutrients deficiencies (7). Although deficiencies are more prevalent after malabsorptive procedures such as bypass, they can also occur in restrictive procedures such as gastric sleeve. According to Oudman et al., the most common bariatric procedure leading to WE is Roux-en-Y gastric bypass, followed by sleeve gastrectomy. Possible mechanisms leading to thiamine deficiency are persistent vomiting, rapid weight loss, poor nutritional intake, malabsorption due to surgical technique, and inadequate vitamin replacement (5).

WE is a neurological syndrome caused by thiamine deficiency (9). It is more common in alcoholics, although any disease that causes malnutrition and thiamine deficiency can give rise to the syndrome (13, 14); for example, cancer patients, gastrointestinal diseases, or gastrointestinal tract surgery lead to a reduction in nutrient intake and absorption (3). Cases of WE following BS have been described for at least 40 years (3). Although this condition is likely underdiagnosed, Chang et al. stated that WE occurs in less than 1% of all patients after receiving BS (15). According to Bathobakae et al., only approximately 15% of all WE cases are diagnosed antemortem due to the ambiguous set of neurological symptoms (9). Olmsted et al., in autopsy studies, revealed that up to 80% of cases of WKS are not diagnosed using the criteria of classical symptoms and signs, such as mental confusion, ocular signs, and ataxia. However, approximately 20% of patients do not present any of these signs, and up to 37% may only present one (16). By chance, our patient presented with the complete triad of mental confusion, nystagmus, and ataxia. The diagnosis of WE remains essentially clinical; the symptoms and signs can be very vague and are mainly related to prolonged post-operative vomiting, rapid weight loss, and an altered mental status (3). WE usually develops in the first 6 months after surgery, although presentation can be earlier if the patient was lower on thiamine before the surgery (9). Our patient was diagnosed 4 months after sleeve gastrectomy surgery, which may indicate that she was lower on thiamine before the surgery. According to Gomes et al., thiamine deficiency seems to affect a significant proportion (15–29%) of preoperative bariatric surgery patients (17). This suggests that obese patients are a risk group for thiamine deficiency, which can be explained by the typical dietary pattern of high consumption of simple sugars and processed carbohydrates (which contain very little thiamine) and low consumption of foods high in thiamine, such as whole grains, legumes, and seeds (17).

There are no reliable and accessible measures to detect thiamine deficiency, despite biochemical changes often preceding overt physical signs (16). Analysis of nutritional status includes 24-h urine excretion of thiamine and blood analysis, such as the thiamine diphosphate test, which measures the level of active thiamine used in biochemical pathways, or erythrocyte transketolase assay, which provides an indirect measure of the most recent utilization of thiamine (10). These analyses have been developed but have limited clinical use due to the processing time required (days to weeks); therefore, they do not offer a practical way of diagnosing WE. It should also be noted that the measurement of serum thiamine is not part of routine laboratory analyses after BS. In our patient, blood thiamine levels were not measured. However, the clinical findings and the effectiveness of the treatment appeared to be sufficient for the diagnosis of WKS. Radiological examination by magnetic resonance imaging (MRI) can be used to confirm the diagnosis of WE (9). A positive test is characterized by hyperintensities in the periventricular areas of the mammillary bodies, thalamus, and periaqueductal and periventricular regions (9, 11). This diagnostic tool has a sensitivity of 50–53% (3, 9, 11) and a specificity of 93% in WE diagnosis (9, 11). Therefore, the absence of hyperintensities is not conclusive (9), and MRI findings are not diagnostic in all cases (3), as was seen in this case report once the brain MRI that was performed did not reveal the typical MRI findings of WE. It is also important to mention that the characteristic lesion sites and signals are not pathognomonic of WE, and consequently, other causes of acute encephalopathy should be considered, for instance, Miller–Fisher syndrome, primary cerebral lymphoma, Bechet’s disease, ventriculoencephalitis, and others (8). Computed tomography (CT) imaging is not the most suitable imaging technique for detecting WE (4). In view of the above, it is important for clinicians to take a good clinical history and be familiar with the risk factors for thiamine deficiency and its signs and symptoms (10). In addition, we argue that clinicians should be extremely attentive to all patients who have even a remote chance of developing nutritional deficiency, for example, patients undergoing obesity surgery.

The normal thiamine requirement for a healthy adult is approximately 1 milligram per day–0.5 mg for every 1,000 Kcal ingested (5). The body stores approximately 30–50 mg of vitamin B1 (8). However, in the case of a deficient diet, severe depletion occurs approximately after 18–20 days of inadequate supply (11). In our case, thiamine reserves were depleted due to anorexia, restricted dietary intake, and low adherence to multivitamin and multimineral supplementation. Thiamine is absorbed in the small intestine, with maximum absorption in the jejunum and ileum (5), preferably in the most acidic environment. Thiamine deficiency can therefore be expected to result from a decrease in acid production (3) as a result of gastrectomy. After being absorbed, this nutrient is transported across the blood–brain barrier by passive and active processes (8).

Thiamine is a water-soluble vitamin (10) that is derived from the diet and is essential for normal brain function (18). This vitamin is required for the metabolism of carbohydrates to produce cellular energy, for the metabolism of lipids for the integrity of the myelin sheath, and for the metabolism of amino acids for the proper synthesis and function of neurotransmitters (3). Depletion of thiamine levels can result in the impairment of thiamine-dependent enzymes, such as pyruvate dehydrogenase and transketolase, causing selective neuronal death (3), which usually leads to lesions that can be seen in MRI (10). In the case of prolonged and severe thiamine deficiency, without adequate replacement, metabolic dysfunction progresses, leading to cerebral lesions and, consequently, cellular death (11).

WE is considered a reversible metabolic disorder that usually responds favorably to therapy with thiamine supplements. However, evidence suggests that therapy may not always lead to complete neurocognitive recovery (18). Without prompt treatment of this condition, WE can progress from an acute, reversible affliction to chronic and more permanent KS, coma, or death (9, 19). There are no standardized national guidelines for the administration of thiamine (16). Although clinicians agree on the empiric treatment for WE, there is no consensus on how much thiamine should be initiated and for how long (9). Despite this, the Royal College of Physician advocates that 500 mg of parenteral thiamine should be given three times daily until symptoms of acute WE resolve (4, 16). The American Society for Metabolic and Bariatric Surgery recommends 200 mg of IV thiamine, three times a day, to 500 mg daily or twice a day for 3–5 days, followed by 250 mg daily for 3 to 5 days or until resolution of symptoms. This is then followed by 100 mg of oral thiamine daily until the patient is no longer at risk of vitamin B1 deficiency (9). The European Federation of Neurological Societies (EFNS) recommends 200 mg, three times per day, until symptoms resolve (20). Our patient was promptly started on thiamine 100 mg IV every 8 h for 4 days and discharged on 100 mg of oral thiamine three times a day. Although there is no consensus on the dosage, frequency, and duration of thiamine administration in the treatment of WE (21), it should be noted that the duration of treatment and the dosage administered were lower than the existing recommendations. Oudman et al., defend that cognitive and motor outcomes are better in groups that receive more than 500 mg/day as an initial dose (14). Additionally, it highlights that longer treatment and higher doses are likely to result in more favorable outcomes (14). According to Infante et al., high doses of intramuscular thiamine for a longer period, with gradual dose reduction after clinical parameters improve, and maintenance of intramuscular thiamine at 200 mg/day for at least 12 months should be employed (21).

It is worth mentioning that oral supplements are not absorbed in significant amounts (4) since oral thiamine has only approximately 5% bioavailability, so considering that IV thiamine is virtually 100% bioavailable, thiamine should be administered IV (10).

The thiamine composition of recommended supplements regarding Bariatric Morango or Citrus, WLS Optimum & Calcium Citrate, and Barovit Sleeve & Calciumplus is shown in Table 1. The supplementation used by our patient was Bariatric Morango, dosed in one chewable capsule a day. Compared to the WLS Optimum & Calcium Citrate and the Barovit Sleeve & Calciumplus, the supplementation used by our patient has a lower dose of vitamin B1, 0.38 mg vs. 2 mg and 1.2 mg, respectively. Consequently, the RDA is also lower, at 32% vs. 167% and 100%, respectively.

**Table 1:** Thiamine content of Bariatric Morango or Citrus, WLS Optimum, and Barovit Sleeve.

Micronutrients	Bariatric Morango or Citrus		WLS Optimum & Calcium Citrate		Barovit Sleeve & Calciumplus	
	Dosage	RDA (%)	Dosage	RDA (%)	Dosage	RDA (%)
Vitamin B1, mg	0.38	32	2	167	1.2	100

RDA, Recommended daily allowance.

Non-users of multivitamin and multimineral supplements may develop poor nutritional status in the long term. According to Oudman et al., 10.3% of WE patients related to obesity surgery showed poor adherence to medication and the follow-up medical regimen (4). Spetz et al. reported an average adherence rate of less than 50% in patients undergoing BS and associated the low adherence reported with side effects, forgetfulness, and difficulty swallowing pills (22). Suggestions for improving patient compliance include reducing the number of tablets, lowering costs, and improving patient and general practitioner education (23). It is therefore important to carry out more studies to identify the need for long-term nutritional monitoring, as well as to consider patients’ barriers to using supplements and understand the best strategies for improving adherence in each case (24).

Since the number of people undergoing BS is increasing, clinicians involved in the surgical treatment of extreme obesity must select patients according to the guidelines. Current guidelines for BS suggest thiamine supplementation for BS patients and a higher dose for patients with suspected thiamine deficiency (4). Prevention is the key since nutritional complications after BS are potentially disabling and may give rise to life-threatening conditions such as WKS. Yet they are often easily preventable (23, 25). Adherence to multivitamin and multimineral supplementation after BS is a crucial determinant of whether complications will occur (25). American and European societies recommend a strict protocol with counseling on supplements and routine assessment of nutrient serum levels in both pre- and post-surgery (23, 25).

Our patient’s initial clinical condition was complex and presented numerous diagnostic challenges, culminating in her going to the emergency department six times before her physical signs of WE were emphasized. She was diagnosed with WE on the 76th post-operative day by a neurologist who observed the classic triad of symptoms, namely confusion, gait ataxia, and ocular dysfunction, including nystagmus. There were no abnormal neuroimaging findings. Thiamine supplement therapy was initiated immediately, and the response was favorable but ultimately not complete.

The patient’s clinical improvement 3 days after starting treatment with parenteral thiamine suggested confirmation of the diagnostic hypothesis of WKS. Early diagnosis and timely treatment are critical to avoid fatal consequences, which is why we emphasize the importance of vitamin B1 dosing in high-risk populations, such as patients undergoing BS. Additionally, we suggest performing effective education on the adverse consequences of malnourishment before the surgery and emphasizing the importance of reinforcing it in the post-surgical period. Exploring nutritional deficiencies in both pre- and post-operative periods is crucial to preventing complications such as WKS (23, 25). Furthermore, we suggest increasing awareness and recognition among health professionals of the need for an early diagnosis and prompt and appropriate treatment in order to avoid sequelae.

## Data availability statement

The raw data supporting the conclusions of this article will be made available by the authors, without undue reservation.

## Ethics statement

This study included only one participant and was conducted in accordance with the Declaration of Helsinki to human studies. The studies were conducted in accordance with the local legislation and institutional requirements. The participant provided written informed consent to participate in this study. Written informed consent was obtained from the participant for the publication of any potentially identifiable images or data included in this article. Written informed consent was obtained from the participant/patient for the publication of this case report.

## Author contributions

MB: Writing – original draft, Formal analysis, Methodology, Investigation. JV: Conceptualization, Formal analysis, Methodology, Writing – review & editing, Investigation. MS: Formal analysis, Writing – review & editing. JC: Conceptualization, Formal analysis, Methodology, Writing – review & editing.
